# Ex Vivo Permeation of Carprofen Vehiculated by PLGA Nanoparticles through Porcine Mucous Membranes and Ophthalmic Tissues

**DOI:** 10.3390/nano10020355

**Published:** 2020-02-18

**Authors:** Lídia Gómez-Segura, Alexander Parra, Ana Cristina Calpena-Campmany, Álvaro Gimeno, Immaculada Gómez de Aranda, Antonio Boix-Montañes

**Affiliations:** 1 Department of Pharmacy and Pharmaceutical Technology and Physical Chemistry, Faculty of Pharmacy and Food Sciences, University of Barcelona, 08028 Barcelona, Spain; lidia.gose@gmail.com (L.G.-S.); anacalpena@ub.edu (A.C.C.-C.); 2 Department of Medicine and Animal Health, Autonomous University of Barcelona, 08193 Bellatera, Spain; 3 Department of Veterinary Medicine and Zootechnic, Faculty of Agricultural Sciences, University of Applied and Environmental Sciences, Bogota RX22+57, Colombia; aleparra@udca.edu.co; 4 Institute of Nanoscience and Nanotechnology (IN2UB), University of Barcelona, 08028 Barcelona, Spain; 5 Department of Animal Research, Animal House of Bellvitge, University of Barcelona, CCiT-UB, 08907 Hospital del Llobregat, Spain; alvarogimeno@ub.edu; 6 Department of Pathology and Experimental Therapeutics, Faculty of Medicine and Health Sciences, Bellvitge Campus, University of Barcelona, 08907 Hospitalet del Llobregat, Spain; igomezdearanda@ub.edu

**Keywords:** nanoparticles, carprofen, solution, drug delivery system, anti-inflammatory, veterinary diseases, NSAIDs

## Abstract

(1) Background: Carprofen (CP), 2-(6-chlorocarbazole) propionic acid, is used as an anti-inflammatory, analgesic and anti-pyretic agent and it belongs to the family of non-steroidal anti-inflammatory drugs (NSAIDs). CP has some adverse reactions in systemic administration; for this reason, topical administration with CP nanoparticles (CP-NPs) can be an optimal alternative. The main objective of this work is the investigation of ex vivo permeation of CP through different types of porcine mucous membranes (buccal, sublingual and vaginal) and ophthalmic tissues (cornea, sclera and conjunctiva) to compare the influence of CP-NPs formulation over a CP solution (CP-Solution). (2) Methods: The ex vivo permeation profiles were evaluated using Franz diffusion cells. Furthermore, in vivo studies were performed to verify that the formulations did not affect the cell structure and to establish the amount retained (*Qr*) in the tissues. (3) Results: Permeation of CP-NPs is more effective in terms of drug retention in almost all tissues (with the exception of sclera and sublingual). In vivo studies show that neither of the two formulations affects tissue structure, so both formulations are safe. (4) Conclusions: It was concluded that CP-NPs may be a useful tool for the topical treatment of local inflammation in veterinary and human medicine.

## 1. Introduction

Non-steroidal anti-inflammatory drugs (NSAIDs) are some of the most commonly used medications in human and veterinary medicine. Carprofen (CP), 2-(6-chlorocarbazole) propionic acid, is used as an anti-inflammatory, analgesic and anti-pyretic agent [1,2]. CP was licensed for systemic human use in several countries in the 1980s and for veterinary use in the 1990s. However, it was withdrawn for human use in the early 1990s for commercial reasons [3,4]. In veterinary medicine, it is still a reference anti-inflammatory and one of the most used in multiple species. The use of CP has been studied in numerous animal species (horse [5,6]; dog [7,8,9]; cat [10]; broiler chicken [11] and rat [12]) however there are no studies in pigs.

The main effect of CP is the inhibition of cyclooxygenase (COX), an important enzyme in the arachidonic acid cascade. This process generates other mediators involved in the inflammatory response, such as prostaglandins and thromboxanes [13]. In veterinary, it is prescribed as a solution for injection or oral tablets for the relief of pain and inflammation associated with osteoarthritis and for the control of postoperative pain associated with soft tissue and orthopedic surgeries in dogs [14]. In addition to osteoarthritis [15,16] and analgesia [17,18] it has been used against respiratory diseases and other pathologies in conjunction with antibiotics [19,20] and as anti-inflammatory after systemic administration [21]. However, some adverse reactions [3] are associated with its systemic administration, and there is no topical formulation commercially available [14]. For this reason, topical administration intended for local action can be an optimal alternative in both veterinary and human medicine [22], majorly concerning mucous tissues where drug permeation is easily suitable [23].

Numerous localized pathologies in pigs could be treated topically such as ophthalmic diseases that occur with inflammation: entropion, distichiasis, trichiasis, lagophthalmia, foreign bodies, wounds, dry keratoconjunctivitis, infections, blepharitis and conjunctivitis [24,25]. Many diseases related to buccal mucous inflammation can be also treated topically: irritations, stomatitis, infectious diseases, autoimmune diseases and fights [26]. Additionally, CP can also treat diseases that cause vaginal inflammation: microbial and parasitic infections, neoplasms, cervicitis, mucometer and subinvolution of placental sites [27,28].

The investigation of most of those pathologies in pigs is reasonably predictive of results in humans. Therefore, the study of anti-inflammatories in this species can provide much information for both veterinary and human medicine.

The suitability of a drug to penetrate through the mucous membrane is correlated with its physicochemical properties, as well as, the pharmaceutical formulation [29,30,31]. On the other hand, it is absolutely necessary the understanding of the processes, pathways and driving forces affecting the transmucosal permeation of the drug [32]. Following these premises, the best way to investigate the dosage of new drugs is in vivo studies. However, in early stages of drug development, in vivo studies are not easy to perform since there are specific ethical and regulatory considerations. Instead, ex vivo studies are an alternative for the investigation of formulations with topical action [33]. Although variations in the experimental setup of diffusion cells could affect the results, some factors (temperature, receptor medium, diffusion area, etc.) can be standardized to obtain consistent and predictive results. Similarly, the availability and acceptability of the mucosal formulations are directly related to the properties of the vehicle/vehicle used [34]. Therefore, characteristics of an ex vivo model to evaluate a new therapeutic agent with localized action on mucous membranes can be very much informative. For example, the comparison of different mucous membranes for the same drug and formulation enhances the robustness of a new animal model to predict topical absorption in humans [35]. Thus, the main goals of this work were the screening of different types of porcine mucous membranes in ex vivo permeation studies of CP, as well as the evaluation of the anti-inflammatory efficacy.

Another aspect to consider when treating a localized topical inflammation concerns formulation effect. It must be ensured that the drug reaches the site of action at therapeutic concentrations and, in addition, these concentrations are maintained for a prolonged time to reduce inflammation. In this sense, the use of nanoparticles (NPs) can be a good alternative to conventional drugs. They represent promising drug carriers for topical applications [36]. For topical treatments in swine, it is necessary to develop drug delivery systems that allow and facilitate the handling of the animal. The slow release nanoparticles such as those consisting of poly D, L-lactide-co-glycolide acid (PLGA) have the advantage that they are more durable over time and therefore do not require as many administrations as systemic conventional solution or tablets.

The main objectives of this work are the investigation of ex vivo permeation of CP through different types of porcine mucous membranes or commonly involved ophthalmic tissues to compare the influence of a PLGA NP formulation [32,36] over a mere drug solution.

## 2. Materials and Methods

### 2.1. Materials

CP was obtained from Capot Chemical (Hangzhou, China) and NPs of CP were prepared as described before [32,36]. Deionized water was obtained from a MilliQ1 Plus System lab. Ketamine (Imalgene^®^) of Merial-Boehringer Ingelheim (Sant Cugat del Vallés, Spain); Xylazine (Rompun^®^) of Bayer Hispania (Sant Joan Despi, Spain); Midazolam Gen^®^ (Midazolam); Propofol Lipuro 1%^®^ (Propofol); Forane^®^ (Isoflurane), Pentothal^®^ (Sodium thiopental) all of them from Centauro Veterinary (Barcelona, Spain). Endotracheal tubes with a low-pressure balloon, number 7 of Centauro Veterinary. All other chemicals were of analytical grade and used without further purification.

### 2.2. NPs of Carprofen

Polymeric NPs of CP were transferred by the team of Parra et al. [36]. Resultant NPs of the study of Parra et al. [36] were freeze-dried and sterilized for more studies. In our investigation, freeze-dried NPs were rehydrated by slow injection of purified water ensuring proper wetting. Then, the vial was gently shaken for 2 min until complete disintegration and dissolution of the content.

#### 2.2.1. Materials of NPs

As explained in the study by Parra et al. [36], the materials used in that study were poly (d,l-lactic-co-glycolic) acid 75:25 (Resomer^®^RG753S, Mw 36,510 Da) and purchased from Evonik Industries (Essen, Germany). Poloxamer 188 (Lutrol^®^F68) was from BASF (Barcelona, Spain). HP-CD was obtained from Sigma-Aldrich (St. Louis, Missouri, USA) and CP from Capot Chemical (Hangzhou, P.R. China). Double distilled water was obtained from a MilliQ^®^ Plus System, lab supplied. All other chemicals were of analytical grade and used without further purification.

#### 2.2.2. Preparation of NPs

As detailed in the study of Parra et al. [36], NPs of matrixial structure (nanospheres) containing CP were elaborated by the solvent displacement technique under the optimized conditions. An organic solution of PLGA (1.98–7.02 mg/mL) in 10 mL of acetone containing CP (0.08–0.92 mg/mL) was poured into 20 mL of an aqueous solution of P188 (5.80–14.20 mg/mL) under moderate stirring and adjusted to pH 3.5. Acetone was evaporated and the NPs dispersion concentrated to 20 mL under reduced pressure (Buchi B-480, Flawil, Switzerland). The resulting NPs were collected by ultracentrifugation (3000 rpm, for 30 min. Sigma 301 K centrifuge, Barcelona, Spain), and washed twice with doubled distilled water [37]. Resultant NPs were freeze-dried and sterilized for in vivo study.

#### 2.2.3. Physicochemical Characterization

The team of Parra et al. [36] described the physicochemical characterization of CP nanoparticles within the following parameters: mean particle size (Z-ave), polydispersity index (PI), zeta potential (ZP) and encapsulation efficiency (EE). The values of each parameter were: Z-ave, which ranged between 176.20 ± 0.36 and 250.17 ± 49.47 nm with narrow distribution; PI values < 0.09 ± 0.09. ZP values ranged from −19.10 ± 1.76 to −26.20 ± 0.46 mV. EE values varied from 74.70 ± 0.95 to 97.10 ± 1.41%.

### 2.3. Permeation Studies

#### 2.3.1. Mucous Membranes

Three different types of porcine mucous were used as permeation membranes: buccal, sublingual and vagina. Additionally, three ophthalmic structures (sclera, cornea and conjunctiva) were also used to evaluate CP permeation and/or penetration. Ex vivo tissues were obtained under veterinary supervision from residual individuals of female pigs (cross Landrace × Large White, 25–30 kg), previously used in surgical university practices and according to the Ethics Committee of Animals Experimentation at the University of Barcelona. Pigs were anesthetized with intramuscular administration of ketamine (3 mg/kg), xylazine (2.5 mg/kg) and midazolam (0.17 mg/kg). After chirurgical experimentation, the animals were euthanized with an intravenous overdose of sodium thiopental. After debridation, samples were frozen to −20 °C and longitudinally cut in 700 µm slabs with a dermatome GA 630 (Aesculap, Tuttlingen, Germany) [38,39,40]. The integrity of the mucous tissues was verified prior to the experiment according to Section 2.4.

#### 2.3.2. Ex Vivo Study: Franz Diffusion Cells

Ex vivo permeation study of NPs was performed in vertical Franz diffusion cells (FDC 400, Crown Glass, Somerville, NY, USA) following a previously validated procedure [41] with a diffusion area of 0.64 cm^2^ for all membranes except for buccal mucous (2.54 cm^2^). A dialysis membrane (Dialysis Tubing Visking, Medicell International Ltd., London, UK) was used according to Parra et al. [36].

Mucous membrane samples were placed between the receptor and donor compartments with the proximal side in contact with the receptor medium and the mucous side in contact with the donor chamber. Samples of 300 µL were placed in the donor compartment (CP-Solution and CP nanoparticles (CP-NPs)). As receptor medium, PBS at pH 7.4 was used and kept at 32 +/− 0.5 °C under continuous stirring in accordance with sink conditions. Samples of 300 µL were withdrawn from the receptor compartment at pre-selected times for 6 h and replaced by an equivalent volume of fresh PBS at the same temperature. This experiment was done in six replicates for each tissue and formulation. At the end of the experiment, samples were analyzed by HPLC–UV.

#### 2.3.3. HPLC–UV

**Figure 1:**
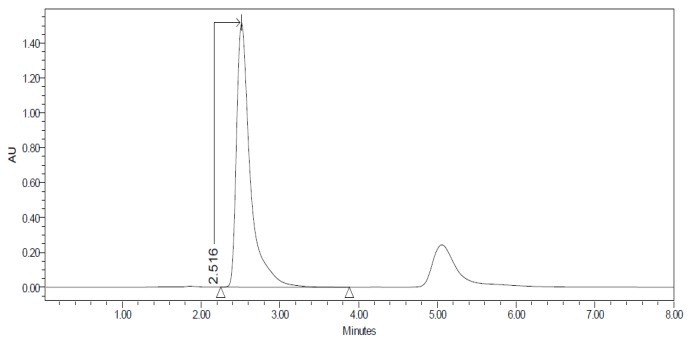
Chromatogram of the Carprofen (CP) validation line at concentration 100 µg/mL.

**Figure 2:**
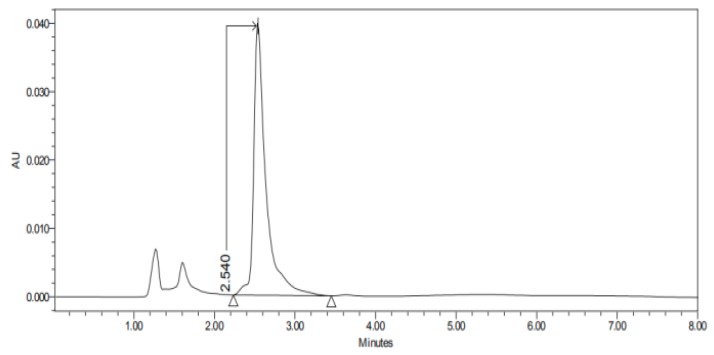
Chromatogram of CP nanoparticles (CP-NPs) in sclera at 6 h.

**Figure 3:**
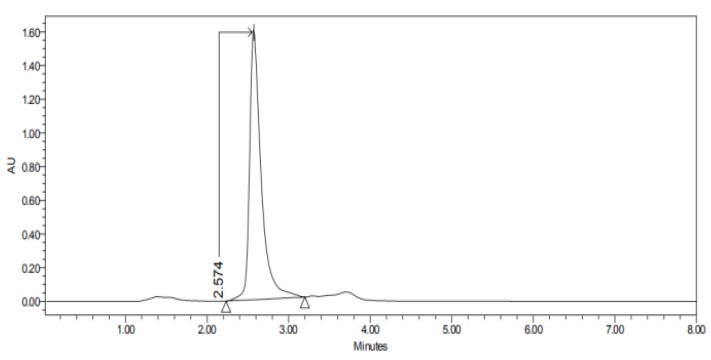
Chromatogram of CP-NPs in cornea at 6 h.

**Figure 4:**
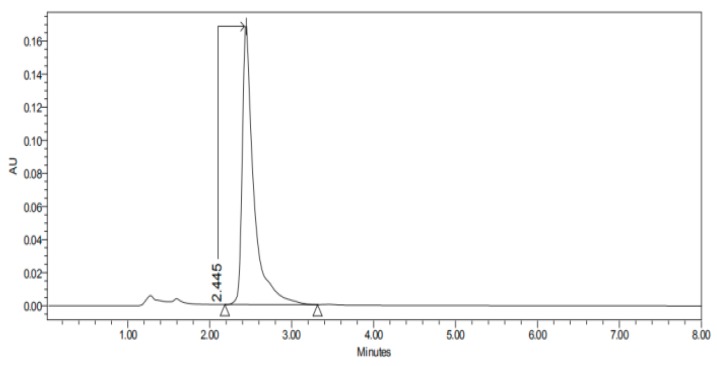
Chromatogram of CP-NPs in conjunctiva at 6 h.

**Figure 5:**
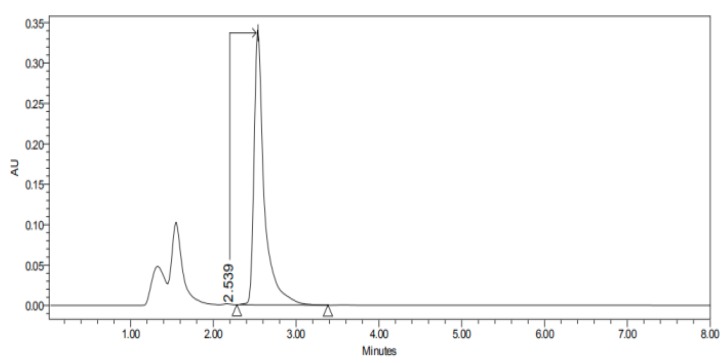
Chromatogram of CP-NPs in buccal mucous at 6 h.

**Figure 6:**
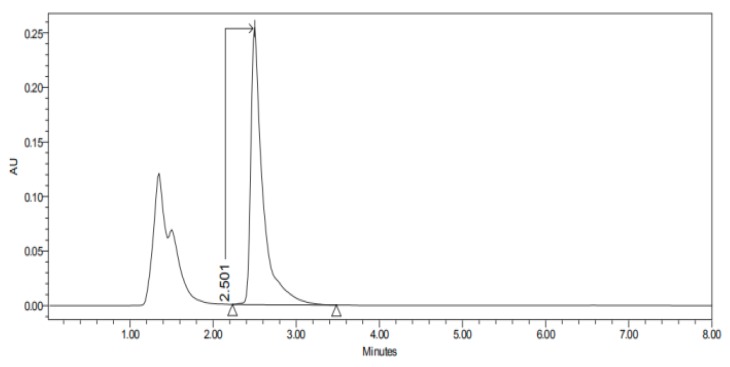
Chromatogram of CP-NPs in sublingual mucous at 6 h.

**Figure 7:**
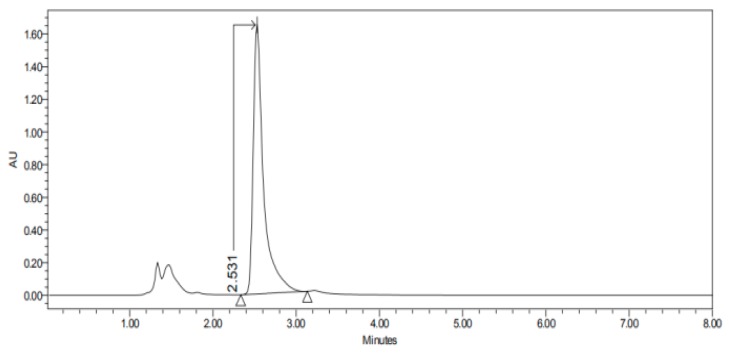
Chromatogram of CP-NPs in vaginal mucous at 6 h.

The HPLC–UV apparatus consisted of a Waters LC Module I plus (Waters Co., Milford, MA, USA), with an ultraviolet detector set up at 235 nm, and Millenium^®^ software (Waters Co., Milford, MA, USA). A reverse-phase column C18, 3.9 × 150 nm packed with 5 µm, was used (Simmetry^®^, Waters Co., Dublin, Ireland). The mobile phase, previously filtered by a 0.45 µm nylon membrane filter (Technokroma, Barcelona, Spain) and degassed by sonication, consisted of methanol and potassium dihydrogen phosphate diluted in 1 L distilled water PH = 3 (75:25). The injection volume was 30 µL and the flow rate was 0.1 mL/min. This method has been validated in our laboratory and is specific and sensitive for the detection of carprofen. The retention times were 2.4–3.6 min as we can see in Figure 1, Figure 2, Figure 3, Figure 4, Figure 5, Figure 6 and Figure 7 and Table 1.

#### 2.3.4. Permeation Parameters

For the permeation assays, the flux values per unit area (*Js* in mg/h cm^2^) across mucous membranes and the permeability coefficients (*Kp* in cm/h) were calculated at the steady state. Lag times (*Tl*) and *Js* values were calculated by linear regression analysis using the Graph Pad Prism^®^ software version 5.01 (GraphPad Software Inc., San Diego, CA, USA) [42]. Stationary flux values followed the following relationship:

(1) Js=Qt/A×t

where *Qt* is the amount of CP which permeates into the receptor compartment (mg). *A* is the active cross-sectional area available for diffusion (cm^−2^) and *t* is the time of exposure (h) per unit area

The permeability coefficient (*Kp*, cm/h) was calculated based on the relationship:

(2) Kp=Js/Co

where *Js* is the flux calculated per unit area at the steady state and *C_o_* is the drug concentration in the donor compartment.

Partition parameter (*P*_1_) and diffusion parameter (*P*_2_) were estimated from the following equations:

(3) Tl=1/6×P2

(4) Kp=P1×P2

The predicted steady-state plasma concentration (*Css*) of drug that would penetrate mucous barrier after topical application, was obtained using the following equation:

(5) Css=(Js×A)/Clp

where *Css* is the plasma steady-state concentration, *Js* the flux/area obtained in this study, *A* the hypothetical area of application and *Clp* the plasmatic clearance. Calculations were addressed on the basis of a maximum application area of 1 cm^2^ and *Cl*p values of 2.18 L/h +/− 0.42.

The retained amount of drug in the tissue (*Qr*, µg/g/cm^2^) was calculated with the following formula:

(6) Qr=(Ex/Px)/A×100/R

where *Ex* (mcg) is the amount of drug extracted, *Px* (g) is the weight of the permeated mucous membranes, *A* (cm^2^) is the active cross-sectional area available for diffusion and *R* is the percentage of recovery of the drug, obtained as described formerly [41].

#### 2.3.5. Statistical Analysis

Nonparametric analysis was performed in these studies because drug permeation through animal tissues follows more closely a log-normal than a normal (Gaussian) distribution [43]. Nonparametric *t*-test (Graph Pad Prism^®^ 5.01, GraphPad Software Inc., San Diego, CA, USA) assessed the significance of the differences between formulations and groups of membranes. A *p*-value < 0.05 was accepted as significant.

### 2.4. In Vivo Studies

Integrity of the mucous tissues after in vivo formulations application was investigated with optical microscopy. The studies were conducted under a protocol approved by the Animal Experimentation Ethics Committee of the University of Barcelona (Spain) with ethical approval code 10619.

Thirteen female pigs (Yorkshire-Landrace) of 45–50 kg were anesthetized for 6 h with a standard sedation protocol based on IM ketamine (Imalgene^®^) at 3 mg/kg; IM xylazine HCl (Rompun^®^) at 2.5 mg/kg and IM midazolam (MidazolamGen^®^) at 0.17 mg/kg. Afterward, anesthesia was induced with IV propofol (Propofol Lipuro 1%^®^) at 2.5 mg/kg from Boehringer Ingelheim, Bayer AG, GES and BBraun, respectively. Maintenance for 6 h was achieved with inhaled isoflurane (Forane^®^ 2%) of Centauro Veterinary administered by tracheal intubation with a low-pressure balloon, number 7.

One female pig was not administered anything (white). CP-Solution was administered to six female pigs and CP-NP was administered to six female pigs (thirteen female pigs in total). Each formulation (CP-Solution and CP-NP) was administered topically and locally in each studied tissue (conjunctiva, cornea, sclera, buccal mucous, sublingual mucous and vaginal mucous) in pigs. The same formulation (CP-Solution or CP-NP) was administered in the tissues studied in the same pig. In summary, *n* = 6 for each biological membrane and formulation and *n* = 1 for each untreated biological membrane. The reason why a single pig was used to collect untreated samples is that we did not expect to find cellular or structural changes in healthy untreated pigs. In this way, the number of animals used is reduced, as indicated in the regulation of 3R (Reduce, Replace and Refine) of animal investigation.

After the in vivo permeation phase, studied porcine mucous and ophthalmic tissues were obtained immediately after the animals were sacrificed by an intravenous overdose of sodium thiopental. Samples were collected and fixed overnight (ON) by immersion in 4% paraformaldehyde (PFA) in phosphate-buffered (PB) 20 mM, pH 7.4 and further processed for paraffin embedding. Vertical histological sections were obtained, stained with hematoxylin and eosin and mounted under a cover slip to be observed at 400× and photographed with a Leica DMD 108 optical microscope. In addition, part of the original tissue was used to calculate the in vivo retained amount (*Qr*) of the drug.

#### Determination of the Amount of Drug Remaining in the Mucous Membrane

At the end of the in vivo study, the mucous membrane specimens were used to determine the amount of retained drug. Mucous and ophthalmic samples were carefully cleaned with gauze soaked in a 0.05% solution of sodium lauryl sulfate, washed with distilled water and blotted dry with filter paper. The permeation area was then excised and weighed. Later, different tissues were perforated using a 30 G needle (BD Ultra FineTM, Beckton Dickinson, Fraga, Spain). Its CP content was extracted with methanol/buffer phosphate solution in an ultra-sonic processor for 20 min (*n* = 6 for each biological membrane and formulation). The resulting solutions were measured by HPLC–UV yielding the amount of CP retained in the membrane (*Qr*, mg/g.cm^2^ of mucous membrane).

## 3. Results

### 3.1. Permeation Studies

The permeation studies were done six replicates for each tissue and formulation. Permeation parameters are summarized in Table 2 and Table 3. Results are expressed as median, maximum and minimum values for each type of membrane and formulation. Significant differences are observed between several membranes, especially conjunctiva, buccal, sublingual and vaginal. In contrast, there are almost no significant differences between the cornea and the sclera. However, other trends have been detected and discussed. In addition, we can see in Figure 1, Figure 2, Figure 3, Figure 4, Figure 5, Figure 6 and Figure 7 the chromatograms and CP-NPs retention times of each tissue are detailed in Table 1.

### 3.2. In Vivo Studies

Histological studies of all the tissues have been carried out in order to verify if the formulations studied do affect the tissue structure and therefore, or its effect is attributable to the drug itself. In all tissues, a blank histological study (without drug), a CP-Solution histological study and a CP-NPs histological study have been performed (*n* = 6 for each biological membrane and formulation and *n* = 1 for each untreated biological membrane).

In the histological photographs of each tissue, the different layers of the untreated membrane are justified (Photos 1, 4, 7, 10, 13 and 15). The mucous membranes are composed of two parts: epithelium (A) and connective tissue (B). Inside the epithelium, the outermost part is the stratified flat keratinized epithelium (A) and the connective tissue is its own laminate (B). These two parts are separated by the basal layer. This is the basic structure of the mucous membranes and each tissue presents its own particularities detailed in the photographs.

## 4. Discussion

### 4.1. HPLC Results

Figure 1, Figure 2, Figure 3, Figure 4, Figure 5, Figure 6 and Figure 7 show the chromatograms of the CP-NPs in each tissue studied and in Table 1 we can see different retention times of each tissue. Figure 1 represents the value of 100 µg/mL in the standard line and its retention time is 2516 min. In the following figures (Figure 2, Figure 3, Figure 4, Figure 5, Figure 6 and Figure 7), the chromatograms of the samples collected at 6 h in each tissue (sclera, cornea, conjunctiva, buccal, sublingual and vaginal, respectively) are represented. As can be seen in Table 1, in all tissues the retention time it is very similar (2445–2574 min.) to the retention time of the standard line (2516 min.). Summarizing, we can say that the method used is very selective and specific for the study of CP.

### 4.2. Ophthalmic Tissues

As we can see in Table 2, in conjunctiva mucous the flow (*Js*) is statistically higher in the CP-Solutions (9.4 mcg/h) than in CP-NPs (4.19 mcg/h). This is interesting, since the flow is the rate of entry into the eye, preventing its residence. So, in this type of mucous, the NPs stay longer in the tissue that is the place of action of the drug. In addition, the *Tl* of the NPs is statistically lower (0.31 h) compared to the results of the CP-Solution (1.45 h). *Tl* indicates the time required to reach a steady state, therefore, the results suggested that CP-NPs are absorbed very rapidly in this tissue and had a high diffusion [44]. In the case of *Kp*, it is statistically higher in the CP-Solution (19.58 cm h) than in the CP-NPs (8.73 cm h). The *Kp* depends a lot on the formula of the drug, and in this case, the NPs have lower permeability, they stay longer in the tissue and can perform their function. Therefore, this may be an indication that NPs can have more effect for a longer time [36]. There are also significant differences between *P*1 and *P*2 between the two types of formulations. *P*1 indicates the distribution between the formulation and the tissue and *P*2 indicates the diffusion of the drug into the tissue. In this case, *P*1 is statistically higher in the CP-Solution formulation (17.01 cm) than in the CP-NPs (2.18 cm). In other words, the distribution between vehicle and membrane is greater in CP-Solutions, and therefore, it penetrates better than CP-NPs. An explanation would be the product formulation itself, since the CP-Solution is dissolved and the CP-NPs are encapsulated [45]. However, *P*2 is statistically higher in the case of CP-NPs (6.32 cm). These results show that although NPs have a shorter lag time penetrating the tissue (probably due to their formulation) [46], once inside, their diffusion and distribution capacity in the tissue is better playing a significant role in its residence inside the conjunctiva. Finally, we can see that there are no significant differences in the *Qr* of the two types of formulations (3.62 mcg/cm^2^/g in CP-Solution and 3.57 mcg/cm^2^/g in CP-NPs). In summary, in the conjunctive mucous, we can say that CP-NPs have advantages, since they have a lower *Js* and *Kp* and a higher *P*2 than the CP-Solution. Therefore, CP-NPs have a lower permeation capacity and a better diffusion inside the tissue.

In cornea, no statistically significant differences are observed for any parameter (Table 2), except for the *Qr*. So, both formulations act in a very similar way. Although there are no differences between *J* and *Kp*, the *Qr* of the CP-NPs (20.89 mcg/cm^2^/g) is statistically greater than in the CP-Solution (16.56 mcg/cm^2^/g). Therefore, we can affirm that CP-NPs are retained in the mucous and therefore have more local activity and are safer (since less drug passes into the bloodstream).

In the sclera, there are no significant differences between the *Js* and the *Kp* between formulations (Table 2). Only statistically differences are in *Tl* and *P*2. In this case, the CP-Solution has a significantly lower *Tl* (2.04 h^−1^) than the CP-NPs (2.7 h^−1^). In addition, the CP-Solution has a statistically higher *P*2 (0.82 cm) than the CP-NPs (0.62 cm). These two parameters suggest that in this tissue the CP-Solution has advantages over the CP-NPs, since CP-Solution takes less time to penetrate the tissue and also diffuses better inside the mucous membrane.

In brief, in the eye, we can conclude that CP-NPs have advantages in the conjunctiva and cornea. In the conjunctiva, CP-NPs take longer to penetrate, so they stay longer in the place of action. In the case of the cornea, although the two formulations act in a similar way, the CP-NPs have a better local activity and are safer. So, CP-NPs are a good option to treat locally inflammatory diseases in these two tissues.

### 4.3. Mucous Membranes

As we can see in Table 3, in the buccal mucous, it can be observed that J and *Kp* are statistically lower in the CP-Solution (0.74 mcg/h in CP-Solution and 0.39 cm in CP-NPs). Therefore, CP-Solution has a lower capacity for permeation and less entry into the bloodstream. However, other factors such as *Tl*, *P*1, *P*2 and *Qr* should be commented on and considered. *Tl* is statistically higher in the CP-Solution (1.65 h) than in the CP-NPs (0.8 h). In addition, *P*1, *P*2 and *Qr* are statistically significantly higher in CP-NPs. A very important factor to consider is that *Qr* in CP-NPs is statistically higher (3.46 mcg/cm^2^/gr) than in CP-Solution (2.38 mcg/cm^2^/gr). Therefore, we can say that CP-NPs are safer and more efficient to treat buccal mucous membranes.

In the sublingual mucous, it can be observed that the flow (*J*) is statistically lower in the CP-NPs (0.31 mcg/h) than in the CP-Solution (4.81 mcg/h) (Table 3). Therefore, as explained before, CP-NPs have a lower bloodstream entry rate, and for this reason, are more effective at the local level. In addition, *Kp* is also statistically lower in CP-NPs (0.65 cm·h) and this factor also makes it a better treatment locally in this tissue. Another factor to take into account is that the *Tl* of the CP-NPs is statistically lower (0.8 h) than that of the CP-Solution (2.77 h), and therefore, it is absorbed into the tissue more quickly. Significant differences are also observed in *P*1 and *P*2. In this case, the P1 is statistically higher in the CP-Solution (16.24 cm); but, the *P*2 is statistically higher in the CP-NPs (2.1 h^−1^) than in the CP-Solution (0.6 h^−1^). Therefore, CP-NPs spread faster inside the sublingual mucous. However, a negative factor is that in this tissue the *Qr* is slightly statistically higher in the CP-Solution (33.14 mcg/cm^2^/g) than in the CP-NPs (29.13 mcg/cm^2^/g). This factor is important, since the higher the *Qr*, the more effective and safer the drug is. However, although the *Qr* is significantly higher in the CP-Solution, the amount of *Qr* in the CP-NPs is also quite high. In summary, taking into account all the factors, we can affirm that CP-NPs are a good candidate for the local treatment of inflammatory diseases.

The last tissue to consider is vaginal mucous (Table 3). In this tissue, we can see that the flow (*J*) and *Kp* are statistically lower in the CP-Solution (3.91 mcg/h and 8.15 cm h, respectively) than in the CP-NPs (8.89 mcg/h and 18.52 cm h, respectively). Therefore, permeation into the bloodstream is lower in the CP-Solution. However, *Tl* is statistically lower in the CP-NPs (1.75 h) than in the CP-Solution (3.34 h). Then, CP-NPs reach a steady state faster than CP-Solution. In addition, *P*2 is statistically higher in CP-NPs (0.99 h^−1^) than in CP-Solution (0.53 h^−1^). So, the diffusion of CP-NPs within the tissue is better. Finally, we can see that the *Qr* in the CP-NPs is much higher (52.81 mcg/cm^2^/g) than in the CP-Solution (23.44 mcg/cm^2^/g). Therefore, the amount of drugs retained in vaginal tissue is higher in CP-NPs, and ultimately, they are safer and more effective.

In summary, we can affirm that CP-NPs have particular advantages in these three tissues. In the sublingual mucous, CP-NPs take longer to penetrate, so they stay longer in the place of action. In addition, CP-NPs are safer and more effective in the buccal and vaginal mucous. So, CP-NPs are a good option to treat locally inflammatory diseases in these three tissues.

### 4.4. In Vivo Studies

When reviewing histological photos, it is important to look at and compare several parameters. The most important part to analyze is the areas that have been in contact with the drug. Therefore, we will focus on the outermost area of the mucous (stratified flat keratinized epithelium and own laminate). On the other hand, it is also important to verify that the cellular structures that make up each tissue do not show alterations [47,48]. Then, we analyze the results in each tissue studied.

**Figure 8:**
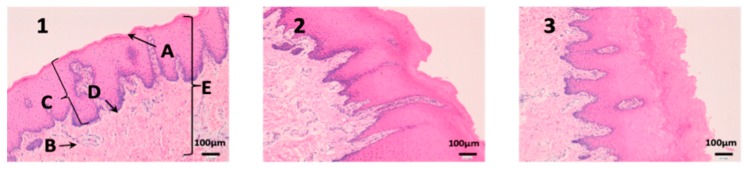
Photo 1: Histological image of untreated buccal mucous observed at 400×; Photo 2: Histological image of buccal mucous treated with CP-Solution observed at 400×; Photo 3: Histological image of buccal mucous treated with CP-NPs observed at 400×. (A) Stratified flat keratinized epithelium; (B) own laminate. (C) dermal papilla; (D) basal layer and (E) buccal mucous.

In buccal mucous (Figure 8) we can see that the outermost part of the tissue (A), and therefore which has been in contact with the drug, is intact in all three tissues and in addition no cell changes are observed. It can be seen that the thickness of the epithelium is different in each photo. This is due to individual variability of the animals and that there are no structural changes caused by the drugs.

**Figure 9:**
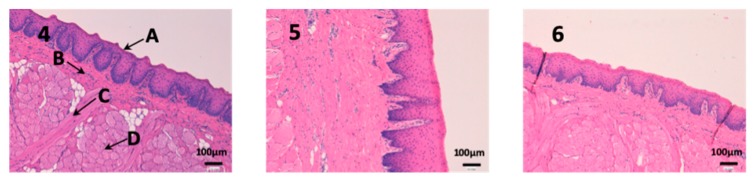
Photo 4: Histological image of untreated sublingual mucous observed at 400×; Photo 5: Histological image of sublingual mucous treated with CP-Solution observed at 400×; Photo 6: Histological image of sublingual mucous treated with CP-NPs observed at 400×. (A) Stratified flat keratinized epithelium; (B) own laminate. (C) muscle and (D) collagen fibers.

Sublingual mucous tissue is a much more muscular tissue than the rest (C) and with much more collagen (D) (Figure 9). However, it is important to highlight that the outermost part of the tissue (A) and cells do not show alterations, and therefore, drugs have not altered the tissue structure.

**Figure 10:**
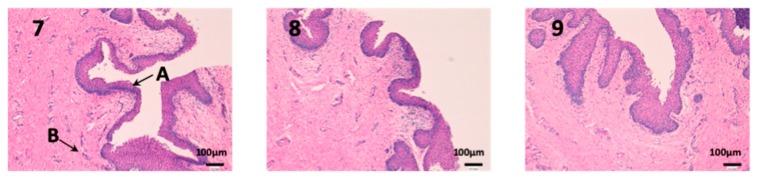
Photo 7: Histological image of untreated vaginal mucous observed at 400×; Photo 8: Histological image of vaginal mucous treated with CP-Solution observed at 400×; Photo 9: Histological image of vaginal mucous treated with CP-NPs observed at 400×. (A) Stratified flat keratinized epithelium; (B) own laminate.

We can see vaginal mucous tissue presents undulations (Figure 10). This is because the pig’s vaginal mucous has invaginations to facilitate intercourse with males. In addition, we can observe differences in the thickness of the epithelium (A) between each photo. These differences are due to the phase of the estrous cycle of the female pig. Depending on the estrous phase in which a female pig is, the thickness of the epithelium is different. Finally, it should be noted that the epithelium (A) is not altered in any of the three photographs.

**Figure 11:**
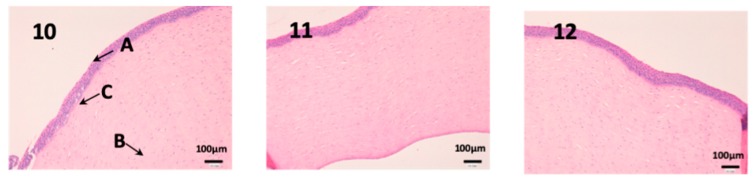
Photo 10: Histological image of untreated cornea observed at 400×; Photo 11: Histological image of cornea treated with CP-Solution observed at 400×; Photo 12: Histological image of cornea treated with CP-NPs observed at 400×. (A) Stratified flat keratinized epithelium; (B) own laminate. (C) Bowman’s membrane.

We can see the structure of the cornea in Figure 11. It is a very fragile and fine tissue. The epithelium (A) and the own laminate (B) are separated by a thin layer called the Bowman membrane (C). As seen in photographs 11 and 12, its structure is not affected and the epithelium (A) and cells are not altered by any drug.

**Figure 12:**
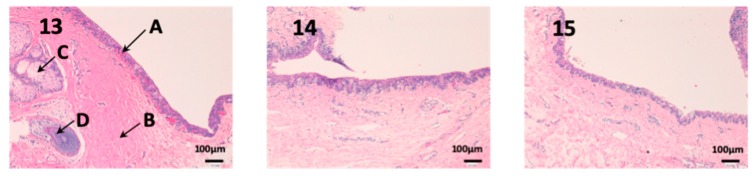
Photo 13: Histological image of untreated conjunctive mucous observed at 400×; Photo 14: Histological image of conjunctive mucous treated with CP-Solution observed at 400×; Photo 15: Histological image of conjunctive mucous treated with CP-NPs observed at 400×. (A) Stratified flat keratinized epithelium; (B) own laminate. (C) oil gland and (D) hair Follicle.

As we can see in Figure 12, the conjunctiva is a very vascularized tissue with oil glands (C). In addition, we can find hair follicles that belong to the eyelashes (D). As seen in photographs eight and nine, neither the epithelium (A) nor the own laminate (B) is affected by any treatment.

**Figure 13:**
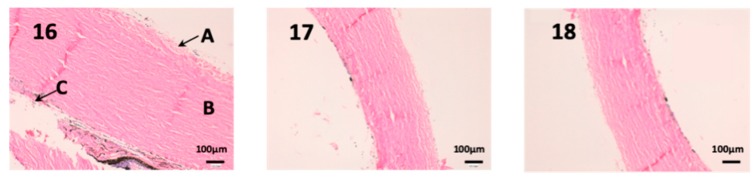
Photo 16: Histological image of untreated sclera observed at 400×; Photo 17: Histological image of sclera treated with CP-Solution observed at 400×; Photo 18: Histological image of sclera treated with CP-NPs observed at 400×. A: Stratified flat keratinized episclera; (B) stroma (collagen fibers). (C) fusca laminate (blood vessels).

In this last figure, we can see the structure of the sclera (Figure 13). This tissue is similar to the cornea, it is very thin, fragile and delicate. The episclera (A) is the outermost layer and its function is to facilitate the sliding of the eyeball with the rest of the eye structures. The stroma (B) is formed by collagen fibers, and finally, the innermost layer is the fusca lamina (C), and it contains abundant blood vessels. As we can see in photographs 14 and 15, no structural part of the sclera is affected by any pharmacological treatment.

Definitely, no histopathological or significant structural changes were observed between control and treated samples. The epithelial cells and the connective tissue beneath showed normal morphology and distribution in the histological mucosal samples analyzed. These results strongly suggest that CP-Solution and CP-NPs do not affect the cellular and tissular morphology and organization in locally in vivo treatment.

## 5. Conclusions

In conclusion, the results show that CP-NPs have advantages in most tissues. They are more effective and safer than the CP-Solution and do not alter the tissue structure. This presents great possibilities for the local treatment of many inflammatory diseases in pigs or humans. In this approach, the side effects of NSAIDs will be minimized. However, additional studies are required to formulate a pharmaceutical presentation that will be easier to apply in pigs to facilitate its administration and animal management.

## Figures and Tables

**Table 1:** Retention times (minutes) of CP-NPs in each studied tissue.

Type of Tissue	Retention Time (Minutes)
CP validation line (100 µg/mL)	2.516
Sclera	2.54
Cornea	2.574
Conjunctiva	2.445
Buccal mucous	2.539
Sublingual mucous	2.501
Vaginal mucous	2.531

**Table 2:** Median, maximum and minimum values of flux (*Js*), lag time (*Tl*), P1, P2, permeability coefficient (*Kp*) and in vivo retained amount (*Qr*) of CP at 6h from the solution of CP (CP-Solution) and nanoparticles of CP (CP-NPs) through ophthalmic membranes (conjunctiva, cornea, sclera).

	CP-Solution			CP-NPs		
	Conjunctiva	Cornea	Sclera	Conjunctiva	Cornea	Sclera
*Js* (mcg/h)	9.4	5.08	0.9	4.19 **	1.24	1.01
	(8.50–10.20)	(1.13–6.16)	(0.22–1.58)	(0.50–7.88)	(0.42–4.73)	(0.96–1.05)
*Tl* (h)	1.45	1.71	2.04	0.31 **	1.72	2.7 **
	(1.42–1.45)	(1.51–2.33)	(1.86–2.22)	(0.19–1.43)	(1.31–2.44)	(2.66–2.73)
*P*2 × 10^1^ (h^–1^)	1.15	0.98	0.82	6.32 **	0.97	0.62 **
	(0.84–1.47)	(0.72–1.10)	(0.75–0.90)	(3.88–8.77)	(0.68–1.27)	(0.61–0.63)
*P*1 × 10^2^ (cm)	17.01	11.91	2.13	2.18 **	3.34	3.39
	(15.80–20.0)	(2.14–15.38)	(0.60–3.66)	(0.12–4.24)	(0.69–9.37)	(3.27–3.50)
*Kp* × 10^3^ (cm·h)	19.58	10.58	1.87	8.73 *	2.58	2.09
	(15.40–22.0)	(2.36–12.83)	(0.45–3.29)	(1.04–16.41)	(0.88–9.85)	(2.0–2.19)
*Qr* (mcg/cm^2^/g)	3.62	16.56	12.21	3.57	20.89 **	12.25
	(3.61–3.63)	(16.10–17.06)	(12.17–12.26)	(2.89–4.25)	(18.55–23.23)	(12.16–12.34)

* *p*-Value < 0.05 and ** *p*-Value < 0.01.

**Table 3:** Median, maximum and minimum values of flux (*Js*), lag time (*Tl*), P1, P2, permeability coefficient (*Kp*) and in vivo retained amount (*Qr*) of CP at 6h from the solution of CP (CP-Solution) and nanoparticles of CP (CP-NPs) through mucous membranes (buccal, sublingual and vaginal).

	CP-Solution			CP-NPs		
	Buccal	Sublingual	Vaginal	Buccal	Sublingual	Vaginal
*Js* (mcg/h)	0.74	4.81	3.91	2.76 **	0.31 **	8.89 **
	(0.73–0.75)	(1.37–8.24)	(3.83–3.99)	(1.70–3.82)	(0.14–0.48)	(5.09–12.69)
*Tl* (h)	1.65	2.77	3.34	0.8 **	2.09 **	1.75 **
	(1.5–1.80)	(2.68–2.87)	(2.57–4.11)	(0.74–0.86)	(1.53–2.65)	(1.4–2.1)
*P*2 × 10^1^ (h^−1^)	1.02	0.6	0.53	2.1 **	0.86 **	0.99 **
	(0.92–1.11)	(0.58–0.62)	(0.41–0.65)	(1.94–2.25)	(0.63–1.09)	(0.79–1.19)
*P*1 × 10^2^ (cm)	0.38	16.24	16.25	0.72*	0.93 **	17.78
	(0.35–0.41)	(4.91–27.56)	(12.82–29.67)	(0.40–1.04)	(0.27–1.60)	(13.35–22.21)
*Kp* × 10^3^ (cm·h)	0.39	10.01	8.15	1.45 **	0.65 **	18.52 **
	(0.38–0.39)	(2.86–17.17)	(7.99–8.31)	(0.89–2.01)	(0.30–1.0)	(10.59–26.44)
*Qr* (mcg/cm^2^/g)	2.38	33.14	23.44	3.46 *	29.13 **	52.81 **
	(2.2–2.56)	(33.09–33.18)	(20.7–26.19)	(2.49–3.63)	(27.81–30.,45)	(49.41–56.21)

* *p*-Value < 0.05 and ** *p*-Value < 0.01.
